# "Borism," or the Toxic Effects of Borax

**Published:** 1896-09

**Authors:** 


ARTICLE III.

### "BORISM," OR THE TOXIC EFFECTS OF BORAX.

Fere ("Sem. Med.") having given borax a six year's trial, in all doses, in epilepsy, finds it far inferior to bromide of potassium in efficacy, and far more dangerous in its toxic effects. Gowers noted the diarrhea, nausea, and vomiting caused even by small doses, and accused it of causing psoriasis. In France early attention was drawn to the digestive troubles and eczema induced by it. The intestinal toxic effects are the most frequent and the earliest. Occasionally absolute intolerance exists, nausea and vomiting occurring after the first dose. More often, after a few doses, there are loss of appetite, sensations of weight and heat at the epigastrium, with ensuing nausea, pains in the temples and vomiting. Administration in glycerine instead of aqueous solution or intestinal antisepsis may remove these effects. Borax determines a peculiar dryness of skin and mucous membranes. The lips and tongue are reddened and denuded of epithelium. The lips and angles of the mouth are fissured, the conjunctivæ injected. The lack of fatty matter in the skin secretion is shown by Arnozan's method. Finely powdered camphor is placed on water, into which is plunged a glass rod previously held in contact with the skin. The camphor particles cease to gyrate if fat be present. No such reaction occurs in "borism." The skin loses its fat in a determined order, naturally sebaceous parts, such as the face, the alae of the nose, being the last affected by borax. Conversely, the fat reappears first in these parts on the stoppage of the drug. The skin dryness, even without an eruption, often coincides witk a dryness of the hair, which falls out. Hence an alopecia of the scalp, which is general. Sometimes even the beard, eyebrows, axillae and pubes are stripped. Constitutional skin rashes may be rekindled by borax, but more special to it are (1) an eczema, like seborrheic eczema, but varied. (a) Papules or small circles, with red and squamous border, which, enlarging, fuse together, forming more or less extended, often symmetrical, plaques. This variety begins in least sebaceous parts, the hypogastrium, lateral regions of trunk, etc. Or (b) eruption of seborrheic acne. with slight desquamation of the scalp, with or without alopecia. These forms usually improve under intestinal antisepsis or local treatment without stoppage of the drug. (c) Pink or red, more or less confluent, plaques, giving the skin a uniform tint, with fine desquamations, deserving the epithet "scarlatiniform." (2) Papular eruptions of variable extent, confluent or not, with pruritus, and followed sometimes by fine branny, sometimes by large flaky, desquamation. Sometimes with the papules are petechiæ. Cachexia, emaciation, edema of face and extremities are often present with these generalized eruptions. Or there may be cachexia without cutaneous lesions, the nails being deeply furrowed transversely, as after fevers. Furuncular outbreaks may occur as accidents of cachexia. A line on the gums, similar to the lead line, is probably microbic. In two patients large, painful, symmetrical "myosites" of the sterno-mastoid muscles were present, with slight rise of temperature; this condition lasted fifteen to twenty days. Lastly, separate swellings of eyelids, or face, or extremities, may occur without any other symptom. The urine will then be found loaded with albumen. As these manifestations may escape notice for a time, the danger is obvious. The cessation of the drug, probably owing to its tardy elimination, by no means always arrests the kidney trouble, as in one case of the author's which went on to uremic coma and death.—*British Medical Journal.*

